# Experimental Data and Modelling of the Solubility of High-Carotenoid Paprika Extract in Supercritical Carbon Dioxide

**DOI:** 10.3390/molecules24224174

**Published:** 2019-11-18

**Authors:** Dorota Kostrzewa, Agnieszka Dobrzyńska-Inger, August Turczyn

**Affiliations:** Łukasiewicz Research Network—New Chemical Syntheses Institute, Al. Tysiąclecia Państwa Polskiego 13A, 24-110 Puławy, Poland; agnieszka.dobrzynska-inger@ins.pulawy.pl (A.D.-I.); august.turczyn@ins.pulawy.pl (A.T.)

**Keywords:** paprika extract, solubility, supercritical carbon dioxide, density-based models, correlation

## Abstract

The studies of solubility of the paprika extract with a high concentration of carotenoids in carbon dioxide under the pressure of 20–50 MPa and at temperatures of 313.15–333.15 K were carried out using the static method. The highest solubility of paprika extract was achieved at the temperature of 333.15 K and under the pressure of 50 MPa. The obtained experimental data were correlated with five density-based models, applied for prediction of solubility in the supercritical carbon dioxide (the Chrastil, del Valle and Aguilera, Adachi and Lu, Sparks et al. and Bian et al. models). The accuracy of particular models with reference to measurement results was specified with the average absolute relative deviation (AARD) and coefficient of determination (R^2^). Results showed that solubility calculated based on the selected models was compliant with experimental data.

## 1. Introduction

Extracts obtained from red paprika (*Capsicum annuum* L.) contain mainly lipid matter (mono-, di- and tri-glycerides, free fatty acids, fatty acid esters), and also essential oils, tocopherol, and carotenoids (mainly capsanthin, capsorubin, zeaxanthin, β-carotene, β-cryptoxanthin) [1,2]. Due to their rich composition, they are widely applied in the food, cosmetic and pharmaceutical industries.

Carotenoids are very important compounds not only for their coloring properties but also for their significant role in the functioning of the human organism [3]. Carotenoids are converted into the active form—vitamin A, which plays a crucial role in vision and the formation of new cells and tissues. The protective effect of carotenoids is related to their antioxidation properties and ability to eliminate free radicals, which damage lipids, proteins and DNA [4]. The increased intake of carotenoids in the diet lowers the content of lipid peroxide in blood plasma, thus protecting against diseases caused by reactive oxygen. Carotenoids reduce the risk of heart attack, stroke, ischemic heart disease, cataracts, age-related macular degeneration, Alzheimer’s disease, cancer, and other age-related diseases [2,3,5].

Traditionally, the paprika extract is industrially obtained using extraction with organic solvents [3]. However, large amounts of hazardous substances which can remain in the final product are used in this method. Moreover, the obtained extract is thermally processed in order to remove the solvent from the extract, which can cause degradation of biologically active compounds contained in the extract [6]. Problems related to the application of organic solvents, consumers’ preferences and legal regulations all increase the interest of using the supercritical fluids in order to obtain natural products of plant origin [7]. The technology using solvents such as carbon dioxide is a real and interesting alternative, as it eliminates drawbacks of extraction with organic solvents and ensures both a very high recovery and quality of the final product [6,8].

The first stage of design and evaluation of all the processes with supercritical fluids is the determination of substance solubility in these solvents [6,7]. This gives information about solvent’s capacity to dissolve and about its selectivity, and it allows us to select the most appropriate parameters for extraction and separation of extract components. Depending on the process in question, high or low solubility can be sought. High solubility is required for supercritical fluid extraction, whereas low solubility is required for separation of the obtained substances.

Solubility in supercritical fluids depends on numerous factors. The process parameters, i.e., temperature and pressure, are very important because they have an impact on physical properties of substances and solvent density [9]. Molecular weight, polarity and the solute volatility have an effect on the substance solubility. The increase in solute vapor pressure increases the solubility and facilitates the removal of the solute [10]. However, the increase in molecular weight and the presence of polar groups in the structure of the solute decreases their solubility in non-polar solvents. The solute solubility in supercritical fluid is often the result of the relative effect of solvent density and solute volatility.

Mathematical modeling is frequently used to describe the solubility with reference to the temperature and pressure applied. Generally, two methods of modelling or predicting the substance solubility in supercritical fluids are used. They involve the theoretical modeling with the use of equation of state and modeling based on the statistical analysis of experimental data using the semi-empirical and empirical equations. Most frequently applied equations of state for modelling the solubility are: Soave-Redlich-Kwong equation, Peng-Robinson equation and Lee-Kesler-Plocker equation. However, the prediction of solubility with the use of these equations requires tedious calculations to be made and determination of critical and thermo-physical properties of the dissolved substance, which are difficult to achieve. Moreover, for compounds with higher molecular weight, equation of state does not provide good conformity with experimental results [11]. The advantage of empirical and semi-empirical models is that they are based on the simple minimization of errors using the method of least squares and that no physical properties of the dissolved substance are necessary. Only data regarding solubility, temperature, pressure, and solvent density are needed. Therefore, these models are commonly applied for predicting the solubility in the specified range of temperatures and pressures [12,13].

Over the last two decades, a number of scientific literature reports dedicated to determination of the solubility of numerous compounds in supercritical carbon dioxide such as drugs, oils, pigments and biologically active compounds have been published [7,14,15,16,17,18,19]. It should be noted, however, that many studies presented in the literature refer to the solubility of pure substances. These results do not reflect all the phenomena occurring during extraction of deferent mixtures. Due to technological reasons, studying the multicomponent solute system is very important as the solubility of the pure compound is largely different to that of the same compound in multicomponent mixture [20]. This can be related to the nature of intermolecular interactions in the supercritical fluid and specific interactions between the solute–solute and solute–solvent molecules.

Although there are literature reports about the solubility of paprika extract in the supercritical carbon dioxide, they mainly refer to extracts obtained from pungency paprika which contain capsaicinoids. The presence of capsaicinoids can have an effect on the behavior and solubility of other substances in the extract. Moreover, because of simplicity, most of the solubility measurements of paprika extracts presented in the literature are based on the dynamic method [21,22,23,24]. These data refer to the apparent solubility of low-carotenoid extracts obtained in the first stage of extraction. Moreover, the data presented are limited to the narrow range of pressures (14–30 MPa).

The aim of this work was to determine the solubility of high-carotenoid paprika extract in the supercritical carbon dioxide using the static method. The solubility studies of paprika extract were carried out in the temperature range: 313.15–333.15 K and pressure of: 20–50 MPa. A very important aspect of analysis was to check the possibility of predicting the solubility with the use of commonly applied models based on density.

## 2. Results and Discussion

### 2.1. Experimental Solubility Results

According to the analytical characterization of paprika extract applied in this work, Table 1 shows characteristics of the compounds present in the extract used for solubility studies.

Table 2 shows the measurement results of paprika extract solubility (S) in carbon dioxide using the static method. Each reported data point is the average of five replicate measurements. The experimental data were expressed as an average value with a standard deviation.

Studies carried out showed that for the applied parameters (pressure and temperature), the solubility of paprika extract in carbon dioxide changed in the ranges of 3.8 g/kg CO_2_ to 8.6 g/kg CO_2_. The standard deviation ranged from 0.25–0.45, and it was related to the specificity of studies carried out under high pressures.

**Figure 1:**
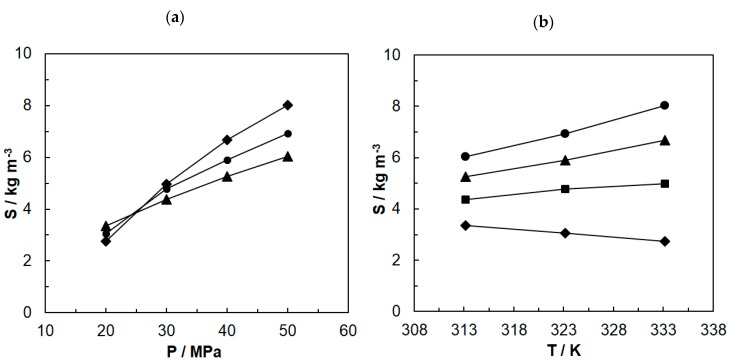
Solubility (S) of paprika extract in supercritical carbon dioxide: (**a**) solubility of extract at various temperatures depending on the pressure ((▲) 313.15 K; (●) 323.15 K; (♦) 333.15 K), (**b**) solubility of extract at various pressures depending on the temperature ((♦) 20 MPa; (■) 30MPa; (▲) 40 MPa; (●) 50 MPa).

Figure 1 shows the solubility curves of paprika extract in carbon dioxide at temperatures of 313.15–333.15 K and under the pressure of 20–50 MPa. The solubility of paprika extract increases with the pressure increase at constant temperature (Figure 1a). This tendency can be explained with the increase in the solvent density and specific interactions between molecules of solute and those of solvent, under the increased pressure.

The effect of temperature is more complex (Figure 1b). Solubility of extract increases with the temperature increase for the pressure of 30–50 MPa when the increase in the vapor pressure of the solute is dominant. For studies carried out under the pressure of 20 MPa, solubility decreases with the temperature increase as a result of decrease in carbon dioxide density. The occurrence of these two competitive effects leads to the crossing of solubility isotherms, broadly described in the literature [10,25,26]. Studies showed (Figure 1a) that an isotherm crossing area for paprika extract solubility falls between 20 and 30 MPa. While comparing the obtained results with literature reports, it can be observed that this pressure is lower than the pressure of 35 MPa obtained for oil from peanuts [27], the pressure of 30 MPa obtained for soya oil [10] and the pressure of 28–34 MPa obtained for oil from pistachio [28]. However, the obtained range of isotherm crossing is similar to results achieved by Fernandez-Ronco et al. for paprika extract with high capsaicinoids content [21]. The existing differences are probably caused by the fact that the discussed oils and paprika extracts are complex mixtures consisting of various components. The nature and content of particular components have an effect on the specific curves of extract solubility in carbon dioxide.

**Figure 2:**
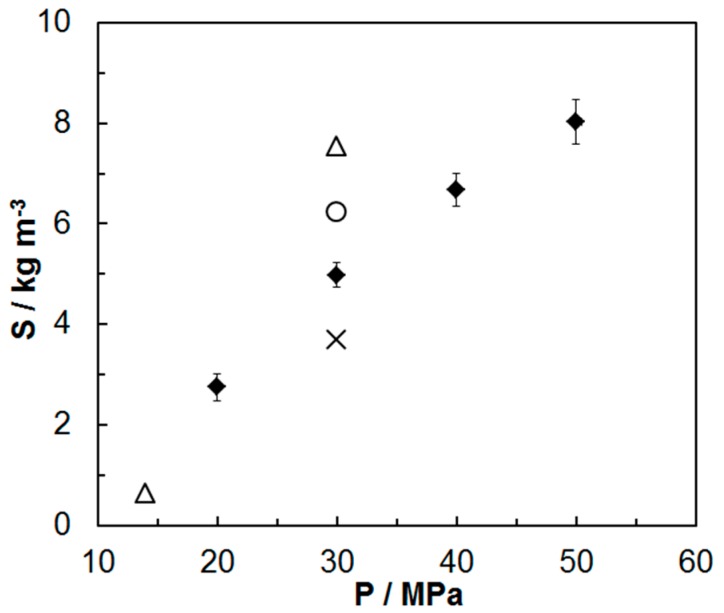
Comparison of experimental data of paprika extract solubility (S) in carbon dioxide at 333.15 K with literature reports: (Δ) paprika extract 2500 CU [21]; (×) paprika extract 140,000 CU [21]; (ο) paprika extract [24]; (♦) results of this work.

The experimental data achieved for paprika extract were compared to available literature data (Figure 2). The results of analyses carried out show that solubility values determined at 333.15 K and for the pressure of 30 MPa vary significantly. Paprika extract solubility determined in this work is lower than solubility determined by Fernández-Ronco et al. and Ambrogi et al. for low-carotenoid extracts [21,24]. However, it is higher than the solubility achieved by Fernández-Ronco et al. for high-carotenoid extract. Differences in solubility may be related to the composition of studied extracts and method for measurement of solubility (i.e., dynamic, static, online or offline) [22]. Solubility determined by Fernández-Ronco et al. [21] and Ambrogi et al. [24] for the dynamic method refers to the apparent solubility of chemically less complex extracts obtained in the first stage of extraction. Therefore, these extracts have better solubility. Simultaneously, it can be observed that the increased carotenoids content in extract reduces the extract solubility. This is due to the fact that these compounds are less soluble in the supercritical fluid than lipid substances present in the extract. The lack of complete characteristics of extracts, for which solubility data were presented in the literature, prevents the exact clarification of the existing differences. All components and their compositions affect the volatility and solubility of the extract. For pressures (40–50 MPa) different than those presented in literature, the obtained data supplement the knowledge of paprika extract solubility in carbon dioxide.

### 2.2. Correlation Results with Experimental Solubility Data

The experimental data obtained in this work were used for calculation of semi-empirical equations coefficients for paprika extract solubility for the studied variables. For modelling, the density-based models proposed by Chrastil, del Valle and Aguilera, Adachi and Lu, Sparks et al. and Bian et al. were applied because of their simplicity and broad applicability.

Chrastil [29] proposed one of the first equations derived from association theory (Equation (1)). This is based on a hypothesis that one molecule of a solute A associates with k molecules of a solvent B to form a solvato complex AB_k_ at equilibrium with the system. There is a linear dependency between solubility logarithm and density logarithm of a supercritical pure fluid and dependency of solubility on the temperature.

(1) $$S=\rho^{k}\exp\left(\frac{A}{T}+B\right)$$

where: S is the solubility of the solute in the solvent (kg m^−3^), ρ is the density of the solvent (kg m^−3^), T is the operating temperature (K). Constant k expresses the average number which describes a number of CO_2_ molecules in the formed complex and it is a characteristic value for a particular system. Constant A is a function of the enthalpy of solvation and enthalpy of vaporization (A = ΔH/RT), R is the universal gas constant (8.314 J mol^−1^ K^−1^), constant B is a function of association number and molecular weight of solute and solvent. Constants k, A, B of equation can be determined using the regression of experimental data.

Chrastil’s equation has certain limits as it is not suitable for solubility higher than 100–200 kg m^−3^ [30]. Chrastil’s model has been modified several times and the obtained equations aimed at improving the conformity of calculations results with experimental data.

The model proposed by del Valle and Aguilera is the modification of the model proposed by Chrastil and it takes into account changes of vaporization enthalpy with temperature (Equation (2)) [31].

(2) $$S=\rho^{k}\exp\left(\frac{A}{T}+B+\frac{C}{T^{2}}\right)$$

The constants A and C represent thermal effects which occur in dissolution process, whereas the constant B is related to molecular weight of the solute.

For both the Chrastil’s model and del Valle – Aguiler’s model, k association number is constant and it does not depend on density. However, Adachi and Lu proposed a new equation in which they made the association number k dependent on solvent density (Equation (3)) [32]. The modification made allows the achievement of the following errors during the prediction of substance solubility.

(3) $$S=\rho^{\left(e_{0}+e_{1}\rho +e_{2}\rho^{2}\right)}\exp\left(\frac{A}{T}+B\right)$$

Sparks et al. combined Adachi-Lu and del Valle-Aguilera equations, and they proposed a six-parameter equation (Equation (4)) [33]. It is recommended for high-pressure applications.

(4) $$S=\rho^{\left(e_{0}+e_{1}\rho +e_{2}\rho^{2}\right)}\exp\left(\frac{A}{T}+B+\frac{C}{T^{2}}\right)$$

Taking into account a non-linear dependency between the solubility and density of supercritical fluid for a wide scope of temperatures and pressures, and also the effect of temperature and density on association number, solvation enthalpy and vaporization enthalpy, Bian et al. proposed another six-parameter empirical equation for the specification of compound solubility in supercritical fluid (Equation (5)) [34].

(5) $$S=\rho^{\left(e_{0}+e_{1}\rho +\frac{e_{2}}{\mathrm{lnT}}\right)}\exp\left(\frac{A+C\rho }{T}+B\right)$$

Consistence and practical value of the above semi-empirical equations for the description of solubility curves were assessed by analysis of determination coefficient value (R^2^) and the value of average absolute relative deviation (AARD). The AARD between the calculated and experimental solubility was determined based on the following equation (Equation (6)):

(6) $$\mathrm{AARD}\;\left(\%\right)=\frac{100}{N}\overset{N}{\underset{i=1}{\sum }}\frac{\left|y_{i,\;\mathrm{calculated}}-y_{i,\;\mathrm{experimental}}\right|}{y_{i,\;\mathrm{experimental}}}$$

where: N is the number of measuring points, y_i, *experimental*_ – solubility determined in experiment i, y_i, *calculated*_ – solubility calculated for experimental point i.

Calculations of coefficients in semi-empirical equations were done with Solver program in Microsoft Excel 2010 using the least squares method. Initial values for every parameter were estimated, and then square sums for every iteration were calculated point after point until convergence tests were completed.

Table 3 presents the results of mathematical modeling of paprika extract solubility in supercritical carbon dioxide with the use of the discussed models. High values of determination coefficients R^2^ (0.9954–0.9983) and low values of average absolute relative deviation AARD (1.15–1.99) indicate that all models describe experimental data with great accuracy.

Values of constant A, which is the function of solvation and vaporization enthalpies (ΔH/R) were very similar in all equations. While comparing these values with literature reports, it can be recorded that these values are similar to the value given for triolein [12]. Based on the determined coefficient A, the value of total dissolution heat for paprika extract was determined for studied temperatures and it was 21.01–21.10 kJ mol^−1^.

Values of association constant k and parameter B in the Chrastil model and del Valle and Aguilera model were identical, whereas constant C was very low in Sparks et al. equation as compared to del Valle and Aguilera equation. Therefore, modification made by del Valle and Aguilera to Chrastil’s equation does not cause significant changes in description of experimental data. However, modification made by Adachi and Lu making the association number k dependent on solvent density had the effect on improving the description of experimental data for the studied range of pressures.

**Figure 3:**
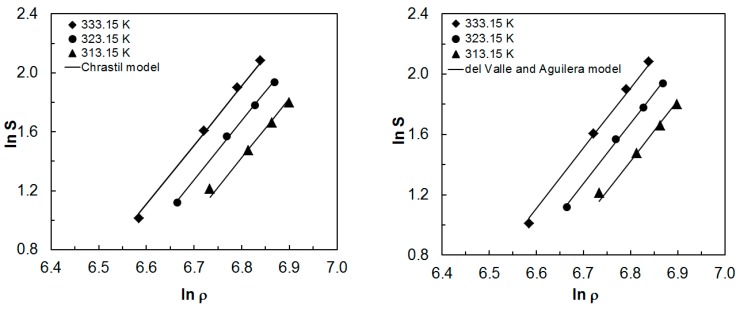

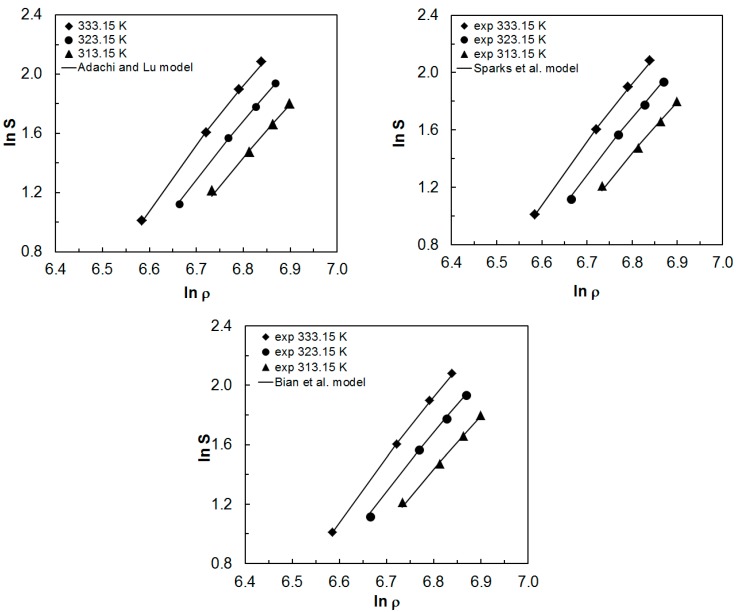
Comparison of the experimental and correlated solubility data.

Vales of constants A, B, e_0_, e_1_, e_2_ in the Adachi and Lu and Sparks et al. model were similar, and constant C in Spark et al.’s equation was also very low. As a consequence, the application of the entire Sparks et al. model with 6 controlled parameters does not improve the accuracy of experimental data description but these models remain in great conformity. Further modifications of the Bian et al. model, which identifies the effect of temperature and density not only on the average number of associations but also on solvation and vaporization enthalpies, lead to only slight improvement of Adachi and Lu’s equation with 5 controlled parameters. Figure 3 shows correlations between experimental data of paprika extract solubility in supercritical carbon dioxide and the values achieved with the use of the discussed equations. The obtained curves are parallel, which indicates a good adjustment for the entire range of temperatures.

The analysis of literature reports showed that the Chrastil, del Valle and Aguilera as well as Adachi and Lu models also described experimental data of pungency paprika extract solubility with high accuracy [21,23,35]. As presented in this work, Fernández-Ronco et al. achieved the best adjustment for Adachi and Lu equation [21]. At the same time, Fernández-Ronco et al. [21] and Illés et al. [23] obtained higher values of the association constant k in the Chrastil equation (10.18 and 11.067 respectively) compared to data achieved in the present work. This can be attributed to the fact that differences in solubility occur mainly in the high pressure region. At low pressure and at low density of carbon dioxide, the solubility determined in our study using the static method are similar to those given by Fernández-Ronco et al. using the dynamic method. The values of parameter A in the Chrastil equation determined by Fernández-Ronco et al. (A = −5226.34) and Illés et al. (A = −4364.72), which takes into account the heat of solvation and vaporization of the solute, were higher than the absolute value achieved in the present work. This indicates that the solubility of extracts for which data are reported in the literature are most affected by the operating temperature.

## 3. Materials and Methods

### 3.1. Materials

The paprika extract obtained from supercritical CO_2_ extraction process (pressure 45 MPa, temperature 323.15 K) carried out at Łukasiewicz Research Network **–** New Chemical Syntheses Institute in Puławy was the material used for these studies. Carbon dioxide (mass fraction purity 0.999) used as solvent was purchased from “Linde Gaz Polska" LLCo. All the organic solvents and reagents had analytical purity or GC purity and were purchased from Sigma-Aldrich and Avantor Performance Materials Poland S.A. Carotenoids analytical standards for qualitative and quantitative analyses were purchased from CaroteNature GmbH and analytical standards of methyl esters of fatty acids were provided by Sigma-Aldrich.

### 3.2. Measuring Equipment and Experimental Methods

**Figure 4:**
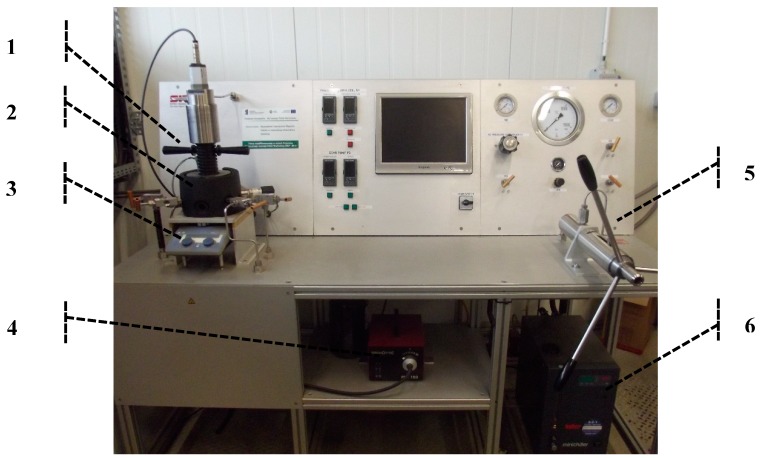
Equipment for solubility and phase equilibrium measurements: (**1**) mobile piston; (**2**) optical chamber; (**3**) magnetic stirrer drive; (**4**) light source for endoscopic specula; (**5**) hand pump; (**6**) cooling thermostat.

Solubility analysis of paprika extract in supercritical carbon dioxide was carried out using the static method. The SITEC (SITEC-Sieber Engineering AG, Maur (Zurich), Switzerland) equipment was applied for carrying out studies (Figure 4). The detailed scheme and description of the equipment applied was presented in the previous paper [18]. The temperature was determined using the PID controller with an uncertainty of 1 K. The pressure was measured with a transducer with an uncertainty of 0.1 MPa.

The known amount of paprika extract (0.4–0.6 g) was placed in the optical chamber with the volume of 30 cm^3^. The chamber was closed and purged with gaseous carbon dioxide in order to remove oxygen. Then, carbon dioxide was introduced to the cell from the bottle up to the pressure of approx. 6 MPa. The content of the chamber was heated to the required temperature and mixed intensely with a magnetic stirrer. After reaching the specified temperature, the pressure in the optic chamber was increased to the required value. When the experience parameters were achieved, mixing was continued for 2 hours and it was turned off and the system was left for 1 hour to reach equilibrium. After this period of time, mixture samples were taken to the sampler with the capacity of 1.5 cm^3^. During sampling, the pressure changes in the scope of ±0.5 MPa were observed.

In order to determine the amount of extract dissolved in carbon dioxide, the content of sampler was expanded to a round-bottomed flask with the capacity of 50 cm^3^, and then the sampler was flushed with acetone (25 cm^3^) to elute the extract. In the subsequent stage, acetone was removed with a rotatory evaporator (R-210, Bȕchi). After being cooled to room temperature, the flask was left in a desiccator to attain solid weight and then the extract weight was measured gravimetrically (±0.0001 g).

### 3.3. Analysis Methods

The chromatographic and spectrophotometric methods were applied for qualitative evaluation of the paprika extract used for solubility studies.

#### 3.3.1. Determination of Total Carotenoid Content

The total content of carotenoids in paprika extract was determined in compliance with the procedure described in Compendium of Food Additive Specifications [36]. This method is based on absorbance measurements of acetone red paprika extract at 462 nm. The absorbance of the sample was measured using JASCO spectrophotometer model V- 650 UV-Vis. The total carotenoid content was calculated according to Equation (7).

(7) $$C_{k}=\frac{100{\;A}_{462}}{2100m_{p}}$$

where: C_k_—total carotenoid content (%), A_462_—sample absorbance at 462 nm, 2100—A^1%^_1cm_ for capsanthin/capsorubin in acetone for 462 nm, m_p_—sample weight (g).

#### 3.3.2. Determination of Extract Color Value

The color value of paprika extract used for studies was determined with the spectrophotometric method [37]. These studies were carried out in compliance with the Official Analytical Methods of the American Spice Trade Association [38]. This method is based on absorbance measurement of acetone paprika extract at 460 nm. The absorbance of the sample was measured using JASCO spectrophotometer model V- 650 UV-Vis. The extract color value was calculated according to Equations (8) and (9).

(8) $$\mathrm{ASTA}=\frac{164{\;A}_{460}f}{m_{p}}$$

(9) 1CU=40.24 ASTA

where: ASTA—American Spice Trade Association color value, CU—Color Units, A_460_—sample absorbance at 460 nm, f—coefficient of correction of optical path length, m_p_—sample weight (g), 164—calculator factor.

#### 3.3.3. Determination of Carotenoids Concentration 

The methodology involved determination of carotenoid content in saponified sample of extract using the internal standard. Waters Liquid chromatograph equipped with DAD 2996 detector and 4.6 × 150 mm chromatographic column YMC^TM^ Carotenoid with granulation of 3 μm which was additionally equipped with a protective column YMC^TM^ Carotenoid S-3, was used for studies. Mixture of methyl alcohol, acetonitrile and water with the ratio of 75:10:15 (Eluent A) in gradient elution with dichloromethane (Eluent B) was used as a mobile phase. The remaining conditions of the chromatographic separation were as follows: flow rate 1 mL/min, injector volume 20 µL, column operation temperature 298.15 K, analysis time 55 min.

In order to specify the content of particular carotenoids in paprika extract, a known amount of extract was dissolved in dichloromethane and the basic solution was obtained. The specified volume of this solution was dissolved in diethyl ether and then it was saponified using 15% KOH. The reaction was carried out in a separator. The formed ether phase was washed with water to obtain neutral pH. The obtained ether solution was dried with anhydrous Na_2_SO_4_. The solvent was removed using the vacuum evaporator and the residual amount was re-dissolved in the specified volume of dichloromethane. The sample thus obtained was filtered by a syringe filter 0.45 μm, and then it was analyzed with HPLC-DAD for wavelength of 450 nm.

The qualitative analysis was based on the comparison of retention times of the obtained peaks with the retention times of peaks obtained for carotenoid standards. The quantitative analysis was carried out using the internal standards method based on the calibration curves. The β-Apo-8’-carotenal, which was also added to real test at the beginning of preparation process, was used as an internal standard. The correlation coefficients of calibration curves for particular carotenoids were R^2^ ≥ 0.995.

#### 3.3.4. Determination of Fatty Acid Content

The method involved indirect determination of the fatty acid (FA) value as methyl esters (FAME) obtained by esterification with trimethylsulfonium hydroxide (TMSH) solution. For the analysis, the Agilent Technologies gaseous chromatograph 6890N coupled with mass spectrometer MSD 5975 was used. Measurements were carried out for the following operating parameters of gas chromatograph: capillary column adjusted to separation of the selected FA: HP-88 (60 m, 0.25 mm i.d., 0.20 μm film thickness); carrier gas flow (He 5.0): from 1.0 to 1.5 mL/min; inject volume: 1 μL; scan range: 50–500 amu; quadrupole temperature: 423.15 K; ion source temperature: 503.15 K; electron energy: −70 eV.

In order to determine the content of fatty acids, a known amount of extract was weighed to reaction vial and 0.5 cm^3^ tert-butyl methyl ether (TBME) was added and mixed. To this solution, 0.25 cm^3^ TMSH solution (0.25 M in methanol) was added and then it was shaken vigorously for 1 min and then it was injected into the chromatographic column.

The qualitative analysis was based on mass spectra available in the NIST Research Library and on the comparison of peak retention times of available standard. Based on calibration curves made, the FAME value was specified and then it was calculated into the FA value in the tested sample.

## 4. Conclusions

This paper shows the experimental data which enhance the knowledge of solubility of high-carotenoid paprika extract in supercritical carbon dioxide. Studies carried out using the static method showed that for the following parameters (20–50 MPa, 313.15–333.15 K), paprika extract solubility varied from 3.8 g/kg CO_2_ to 8.6 g/kg CO_2_. The extract solubility under the pressure of 50 MPa (T = 333.15 K) was twice as high as solubility under 20 MPa (T = 333.15 K).

Based on the achieved data, the relationships between paprika extract solubility and the properties of supercritical carbon dioxide were developed. Paprika extract solubility was correlated with five semi-empirical density-based models: the Chrastil, del Valle and Aguilera, Adachi and Lu, Sparks et al. and Bian et al. The modeling results show that modifications made by Adachi and Lu had the effect on the improvement of AARD compared to Chrastil and del Valle and Aguilera models. For the studied range of parameters, Sparks et al.’s equation does not improve accuracy of the experimental data compared to Adachi and Lu’s equation. In contrast, Bian et al.’s model, which takes into account the effect of temperature and density on the average association number as well as on solvation and vaporization enthalpies, presents only a slight improvement of mapping the experimental data compared to Adachi and Lu’s model. Simultaneously, all the applied models described experimental data with a great accuracy.

## Figures and Tables

**Table 1:** Characteristics of paprika extract.

Components/Parameters	Value
Extract color value (ASTA)	2500.00
Carotenoids content (%)	7.33
Violaxanthin (%)	0.22
Capsorubin (%)	0.70
Capsanthin (%)	5.04
Zeaxanthin (%)	0.42
β-cryptoxanthin (%)	0.20
β-carotene (%)	0.64
Palmitic acid (C16:0) (%)	11.55
Stearic acid (C18:0) (%)	2.11
Oleic acid (C18:1) (%)	9.08
Linoleic acid (C18:2) (%)	48.53
Behenic acid (C22:0) (%)	0.21
Arachidic acid (C20:0) (%)	0.27
α-Linolenic acid (C18:3) (%)	3.16
Lauric acid (C12:0) (%)	0.40

**Table 2:** Solubility (S) of paprika extract in supercritical carbon dioxide ^a^.

T (K)	P (MPa)	ρ ^b^ (kg m^−3^)	S (g kg^−1^)
313.15	20	839.81	4.00 ^c^ ± 0.28 ^d^
	30	909.89	4.80 ± 0.31
	40	956.07	5.50 ± 0.25
	50	991.30	6.10 ± 0.38
323.15	20	784.29	3.90 ± 0.26
	30	870.43	5.50 ± 0.29
	40	923.32	6.40 ± 0.35
	50	962.45	7.20 ± 0.41
333.15	20	723.68	3.80 ± 0.27
	30	829.71	6.00 ± 0.25
	40	890.14	7.50 ± 0.32
	50	933.50	8.60 ± 0.45

^a^ The standard uncertainties, u, of T, P and S are u(T) = 1 K, u(P) = 0.1 MPa and u(S) = 0.14 kg m^−3^. ^b^ Data was taken from NIST Chemistry WebBook (http://webbook.nist.gov/chemistry, 2018). ^c^ Average values taken from five runs. ^d^ ± Uncertainties refer to standard deviation.

**Table 3:** The fitting constants obtained for five semi-empirical density-based correlations.

Parameters	Equation				
	Chrastil	del Valle and Aguilera	Adachi and Lu	Sparks et al.	Bian et al.
k	4.01	4.01	-	-	-
A	−2538.33	−2537.98	−2527.27	−2529.23	−2529.92
B	−17.74	−17.74	−66.20	−65.38	−36.82
C	-	−1.23 × 10^−4^	-	−6.52 × 10^−3^	−1.08
e_0_	-	-	12.62	12.47	4.78
e_1_	-	-	−2.11 × 10^−3^	−2.06 × 10^−3^	−7.34 × 10^−5^
e_2_	-	-	5.02 × 10^−7^	4.85 × 10^−7^	14.69
AARD (%)	1.99	1.99	1.20	1.20	1.15
R^2^	0.9954	0.9954	0.9982	0.9982	0.9983
